# In Vivo Pharmacokinetic Analysis Utilizing Non-Targeted and Targeted Mass Spectrometry and In Vitro Assay against Transient Receptor Potential Channels of Maobushisaishinto and Its Constituent Asiasari Radix

**DOI:** 10.3390/molecules25184283

**Published:** 2020-09-18

**Authors:** Takashi Matsumoto, Mikina Takiyama, Shou Sanechika, Akiko Nakayama, Katsuyuki Aoki, Katsuya Ohbuchi, Hirotaka Kushida, Hitomi Kanno, Akinori Nishi, Junko Watanabe

**Affiliations:** 1 Tsumura Kampo Research Laboratories, Kampo Research & Development Division, Tsumura & Co., Ibaraki 3001192, Japan; takiyama_mikina@mail.tsumura.co.jp (M.T.); sanechika_shou@mail.tsumura.co.jp (S.S.); nakayama_akiko@mail.tsumura.co.jp (A.N.); oobuchi_katsuya@mail.tsumura.co.jp (K.O.); kushida_hirotaka@mail.tsumura.co.jp (H.K.); kanno_hitomi@mail.tsumura.co.jp (H.K.); nishi_akinori@mail.tsumura.co.jp (A.N.); watanabe_junko@mail.tsumura.co.jp (J.W.); 2 Botanical Raw Materials Research Laboratories, Botanical Raw Materials Division, Tsumura & Co., Ibaraki 3001192, Japan; aoki_katsuyuki@mail.tsumura.co.jp

**Keywords:** maobushisaishinto, Asiasari Radix, pharmacokinetics, mass spectrometry, pharmacology, transient receptor potential channel

## Abstract

The Japanese traditional medicine maobushisaishinto (MBST) has been prescribed for treating upper respiratory tract infections, such as a common cold. However, its mode of action is poorly understood, especially concerning the MBST constituent Asiasari Radix (AR). In this study, we focused on AR, with an objective of clarifying its bioavailable active ingredients and role within MBST by performing pharmacokinetic and pharmacological studies. Firstly, we performed qualitative non-targeted analysis utilizing high-resolution mass spectrometry to explore the bioavailable ingredients of AR as well as quantitative targeted analysis to reveal plasma concentrations following oral administration of MBST in rats. Secondly, we performed in vitro pharmacological study of bioavailable AR ingredients in addition to other ingredients of MBST to confirm any agonistic activities against transient receptor potential (TRP) channels. As a result, methyl kakuol and other compounds derived from AR were detected in the rat plasma and showed agonistic activity against TRPA1. This study suggests that methyl kakuol as well as other compounds have the potential to be an active ingredient in AR and thus presumably would contribute in part to the effects exerted by MBST.

## 1. Introduction

Many people suffer morbidity from various upper respiratory tract infections. In most cases, such symptoms are mild and self-limited; however, increased severity of these infections can occur, especially in elderly or pregnant individuals whose immune systems are weakened [1,2]. Viruses, bacteria, fungi, and helminths all represent pathogens infecting the upper respiratory tract. Although a large number of remedies against such infections have been marketed to this date, they may not always result in positive clinical outcomes due to inappropriate treatment regimens or administration. For example, the inadequate dosing of antibiotics, such as their overuse and misuse, has been identified as a significant driver in the emergence of resistant bacteria [3,4,5] or side effects (e.g., gastrointestinal dysfunction, drug-induced eruption, or liver dysfunction) [6,7]. The World Health Organization has for the first time released a list of drug-resistant bacteria that pose the greatest threat to human health [8]. Therefore, demands for the development of appropriate remedies for the treatment of infectious diseases are expected [9].

According to past reports, several herbal medicines have been considered as effective for the treatment of infectious disease because herbal medicines exhibit direct bacteriostatic effects and/or stimulate or recruit the host’s innate immune system [10,11,12]. Maobushisaishinto (MBST) is a traditional Japanese Kampo medicine (known as Ma-Huang-Fu-Zi-Xi-Xin-Tang in Chinese medicine) that has long been used for treating common upper respiratory tract infections (e.g., the common cold). This medicine has been approved for medical use by the Japanese Ministry of Health and Welfare. Iwasaki et al. (2004) conducted a randomized control study of on the extracted granule of MBST in 18 elderly subjects having received the influenza vaccine and claimed that MBST had adjuvant effects 4 weeks following vaccination [13]. Although adjuvant effects were not observed in another clinical study that used a capsule with different formulation from the one used in the study of Iwasaki et al. [14], some basic studies have supported the potential adjuvant effect of MBST. For example, previous studies using mice showed that the administration of MBST increases the production of the titer of antibodies against influenza, especially in older mice [15,16]. On the other hand, Kamei et al. (2000) reported a case study showing that MBST remitted inflammatory markers and fever; in brief, fever and C-reactive protein levels returned to normal in an elderly patient even though antibiotics did not improve the outcome [17]. Results of these clinical studies and some preclinical evidence [18,19,20,21] indicate that MBST may be effective against upper respiratory tract infection because of its anti-inflammatory effect and adjuvanticity.

MBST is composed of the three crude drugs as follows: Ephedrae Herba (prepared from the stem of *Ephedra sinica* Stapf, *Ephedra intermedia* Schrenk et C. A. Meyer, or *Ephedra equisetina* Bunge), Aconiti Radix Processa (prepared from the tuberous root of *Aconitum carmichaeli* Debeaux or *Aconitum japonicum* Thunberg), and Asiasari Radix (AR; prepared from the root and rhizome of *Asiasarum sieboldii* F. Maekawa or *Asiasarum heterotropoides* F. Maekawa var. *mandshuricum* F. Maekawa). The pharmacological actions of MBST are presumably because of the combined actions of each crude drug contained therein. Ephedrae Herba exerts an anti-inflammatory effect as well as bronchodilation [22,23]. Ephedrine and its related ingredients, which are the major ingredients within Ephedrae Herba, have been reported to be absorbed into the body and contribute to the pharmacological actions of Ephedrae Herba [24,25]. Aconiti Radix Processa has an analgesic effect [26], and its bioavailable ingredient neoline as well as other aconitine alkaloids (e.g., ignavine) are considered to contribute to the effect [27,28]. Regarding AR, the root of herbal plants of analogous species with AR has been reported to have an anti-inflammatory [29], analgesic [30], and antimicrobial effects [31,32]. However, its bioavailable active ingredients and pharmacological roles in MBST remain poorly understood.

This study aims to identify bioavailable active ingredients and pharmacological roles of AR within MBST as a remedy for upper respiratory tract infection. For our strategic approach, we performed two-step experiments, with in vivo pharmacokinetic studies utilizing non-targeted and targeted mass spectrometry (MS) analysis as the first step and in vitro pharmacological study as the second. Occasionally, in vitro pharmacological experiments involving herbal medicine are conducted by direct exposure to rodent or human cell lines. However, this method might be unsuitable for understanding the precise action of medicines, as many ingredients within herbal medicines are not absorbed as their intact form [33]. Therefore, we performed a pharmacokinetic study utilizing the non-targeted analysis to explore the bioavailable ingredients of AR using rats, prior to performing the pharmacological study. Non-targeted analysis using liquid chromatography-high-resolution mass spectrometry (LC-HRMS) represents a powerful tool for multicomponent herbal medicine analysis. Furthermore, we quantified the plasma concentrations of bioavailable AR compounds using liquid chromatography-triple quadrupole mass spectrometry (LC-MS/MS). After pharmacokinetic analyses, we performed in vitro pharmacological studies of bioavailable AR ingredients in addition to other ingredients of MBST to identify the active ingredients. Here, we focused on the activity at transient receptor potential (TRP) channels related to the regulation of pain, body temperature, blood flow, and immune function. We selected TRP channels based on reports that AR components have a pungent flavor upon human intake of AR [34] and that some herbal medicine-derived pungent components (e.g., 6-shogaol and cinnamaldehyde) have the potential to activate TRP channels [35]. Additionally, we examined the anti-inflammatory effects of bioavailable AR ingredients through the investigation of the inhibitory effect against lipopolysaccharide (LPS)-induced excessive nitric oxide (NO) production in RAW264.7 cells.

## 2. Results and Discussion

### 2.1. Qualitative (Non-Targeted) Analysis of AR Compounds in Plasma after AR or MBST Administration

**Figure 1:**
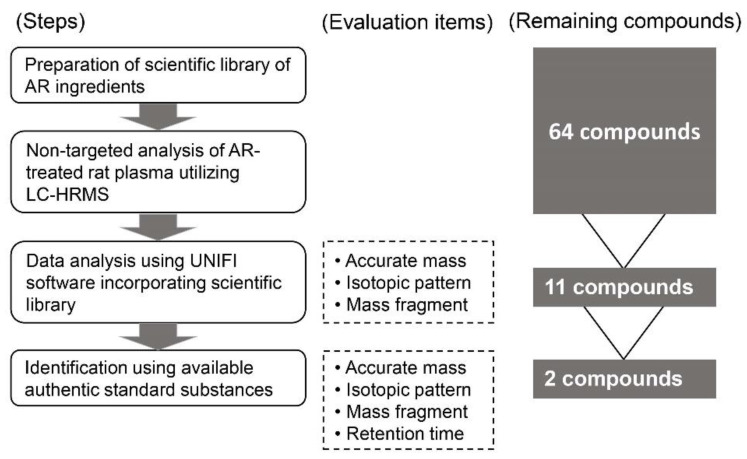
Procedures for liquid chromatography-high-resolution mass spectrometry (LC-HRMS) (non-targeted) analysis.

Non-targeted analysis utilizing LC-HRMS is useful within the pharmacokinetic study of multicomponent products such as those of Kampo medicine, because this analysis enables the acquisition of data for a vast number of components absorbed following drug administration via plasma analysis. Therefore, in this study, we adopted a non-targeted analysis to explore and identify bioavailable AR ingredients. The analysis proceeded in multiple steps (see Figure 1). First, before sample analysis, we prepared an original database that included information about chemical structure, monoisotopic mass, etc., for AR ingredients. Next, we analyzed data from the non-targeted analysis of plasma samples using UNIFI software (Waters, Milford, MA, USA), which is an integrated informatics platform that incorporated the database into a streamlined workflow aimed at identifying chemical components from complex raw data. In our previous non-targeted analysis of plasma following the administration of a natural product, we utilized a well-established database (e.g., kegg compound database); however, many candidate compounds, not only phytomedicines but also endogenous substances (which have same chemical formula and monoisotopic mass), were assigned to one detected peak. Consequently, we could not identify which peaks represented accurate compounds. This incident is widely considered problematic when performing non-targeted analyses of natural products, although researchers have attempted several assign methods or used high-performance technologies such as multiple-stage mass spectrometric analysis. To resolve this problem, we made an original database of 64 AR ingredients based on the Kampo database [36], articles [29,37], and a book [38], which reported ingredients contained in AR (see the list of 64 ingredients shown in Table S1). We believe that this method of non-targeted analysis is quite useful for identifying bioavailable ingredients in natural products.

Since MBST is an oral formulation, AR and MBST were administered orally to rats, and plasma samples were analyzed via non-targeted analysis utilizing LC-HRMS. Because the rat plasma collected at 1 h following the administration of MBST was used in both the qualitative (non-targeted) analysis as well as the quantitative (targeted) analysis that was mentioned afterwards, the sample number was set to three in order to conform to the interindividual difference of the values of the quantitative analysis. On the other hand, the rat plasma following the administration of AR was only used in the qualitative (non-targeted) analysis. The amount of AR ingredients per dose was higher in the dose of 2 g/kg AR compared with the dose of 2 g/kg MBST because MBST is a mixture of three crude drugs. Therefore, the sample number (n = 1) of the AR-treated rat plasma was considered to be enough for the exploration of AR ingredients in the plasma. As shown in Table 1, 11 peaks detected and 8 compounds assigned within rat plasma were estimated to be components derived from AR. The structural formulas of the eight compounds are shown in Figure S1. Among the eight compounds, estragole and (7α,7′β,8α,8′α)-3,4-methylenedioxy-3′,4′-dihydroxy-7,9′:7′,9-diepoxylignane were only detected in the AR-treated rat plasma. A possible reason to be considered for this is that both of the detected compounds might be below the detection limit in the MBST-treated rat plasma because the amount of AR ingredients per dose was lower in case of the dose of 2 g/kg MBST compared with the dose of 2 g/kg AR. The assessment on whether the detected peaks represented AR components was based on accurate mass and fragment information. Subsequently, among the estimated ingredients, two were identified to be methyl kakuol and (2*E*,4*E*,8*Z*,10*E*)-*N*-isobutyl-2,4,8,10-dodecatetraenamide (amide A) by comparing the analytical findings (i.e., retention time, monoisotopic mass, and fragment ions) of their authentic standard substances. The MS and tandem MS (MS/MS) information used for identifying these two compounds are summarized in Figure S2. Other peaks assigned as AR compounds in the non-targeted analysis were not identified due to a lack of available standard substances. Meanwhile, (2*E*,4*E*,8*Z*,10*Z*)-*N*-isobutyl-2,4,8,10-dodecatetraenamide (amide B) and *N*-isobutyl-2,4,8,10-dodecatetraenamide isomer were tentatively identified based upon analytical information using an LC separation with a conventional octadecylsilyl column of ingredients in *Echinacea* Plant Materials and Dietary Supplements [39]. Although asarinin and sesamin are known as orally absorbable compounds and representative ingredients within AR [40], they were not detected in the plasma of rats treated with AR or MBST.

The metabolites of methyl kakuol and amide A have been also confirmed in this study, which could be measured by non-targeted analysis. The results of the exploration of the metabolites formed by phase I metabolism (oxidation, methylation, dehydrogenation, alkylation, water adduction, etc.) and phase II metabolism (glucuronide conjugation, sulfate conjugation, etc.) suggested that methyl kakuol is metabolized into a sulfate conjugate and a dehydrogenated + sulfate conjugate form, as well as amide A is metabolized into an alkylated and a water adducted + alkylated form. Their peaks were detected in AR- or MBST-treated plasma, but not in the extract of AR or MBST. Although sesamin could not be measured by non-targeted analysis, it was reported in a previous study to be metabolized into various forms, such as sesamin monocatechol, sesamin dicatechol, sulfate conjugate, and a glucuronide conjugate [41]. Therefore, the metabolites of AR ingredients might also be responsible for the pharmacological activities of AR and MBST. Further studies (e.g., the organic synthesis of the metabolites, the identification of the metabolites in the plasma, as well as pharmacokinetic and pharmacological studies of the metabolites if the metabolites could be identified in the plasma) to elucidate this possibility are needed in the future. This study has revealed for the first time that some compounds (methyl kakuol, amide A, amide B, etc.) are absorbed into the blood following oral AR administration. Li et al. (2014) reported that 47 components partially corresponding to the ingredients detected in our study were detected by gas chromatography-mass spectrometry and LC-HRMS analysis using rabbit plasma and cerebrospinal fluid following internasal administration of AR [37]. Among the 47 compounds detected in the report, some detected by LC-HRMS analysis, such as kakuol, spilanthol, and xanthoxylol, were not detected in our study. Because drugs administered through the nasal route do not undergo first-pass effects, unlike oral administration, only those compounds detected in plasma following nasal administration can be thought to have a high susceptibility to first-pass effects.

Analytical methods (e.g., LC mobile phase, column, and mass parameters) in association with the non-targeted analysis were selected based on our experiences as well as previous report [42]. To enable the non-targeted analysis to detect more extensive compounds in the plasma, the pretreatment method was selected to be the deproteination method. A matrix effect in the detection by the non-targeted analysis was evaluated in regard of the methyl kakuol and amide A identified in the AR- or MBST-treated rat plasma, using three different rat blank plasma. Consequently, the matrix effects (peak area ratio of the analytes in the solution with plasma/without plasma) in the detection of methyl kakuol and amide A were observed to be 96.8% and 202.8%, respectively. Therefore, it is possible that the persisting components after the pretreatment of the plasma have either a little or an enhancing effect on the ionization of the methyl kakuol and amide A among the AR ingredients. As mentioned above, asarinin and sesamin in AR- or MBST-treated rat plasma were not detected during this non-targeted analysis, although a previous study reported the detection of both compounds [40] as well as the targeted LC-MS/MS analysis that was mentioned afterwards. Because neither asarinin nor sesamin was detected by the non-targeted analysis—even during the analysis of the extracted sample of AR without pretreatment—it was considered that the reason is attributable to the parameters of the LC or mass spectrometer but not to the pretreatment method. Our targeted analysis as well as another study [40] which optimized the analytical methods of asarinin and sesamin used ammonium acetate solution as the LC mobile phase due to the sensitivity of the ammonium adduct ions ([M + NH^4^]^+^, *m*/*z* 372.1) being higher than that of other adduct ions in the MS/MS detection. On the other hand, the LC mobile phase of the non-targeted analysis was composed of 0.1 vol% formic acid and acetonitrile. Taken together, it is considered that neither asarinin nor sesamin was detected in the non-targeted analysis, as there was no ammonium adduct ion produced due to no ammonium source in the LC mobile phases being incorporated within the non-targeted analysis.

### 2.2. Quantitative (Targeted) Analysis AR Ingredients in Plasma after MBST Administration

To determine the plasma concentrations of AR compounds identified within the non-targeted analysis, we performed a quantitative LC-MS/MS analysis of plasma from MBST-treated rats using selected reaction monitoring (SRM) mode, which is suitable for the highly selective and sensitive analysis of the target compound. As described above, asarinin and sesamin were not detected in AR-treated rats via non-targeted LC-HRMS analysis, although a previous report found that both are absorbed into the blood following oral administration of AR [40]; therefore, asarinin and sesamin concentrations were also measured within the plasma. The representative SRM chromatograms of methyl kakuol, amide A, asarinin, and sesamin in the blank plasma with a mixture of their standard substances, as well as blank plasma, and plasma collected at 1 h after the administration of 1 g/kg of MBST, are summarized in Figure S3. All the calibration curves were prepared by a high correlation coefficient (r > 0.996) and the accuracy of each point of the calibration curve was in the range of 88.4%–111%, suggesting that there is a high quality of the determination of AR ingredients in the plasma used in this study.

**Figure 2:**
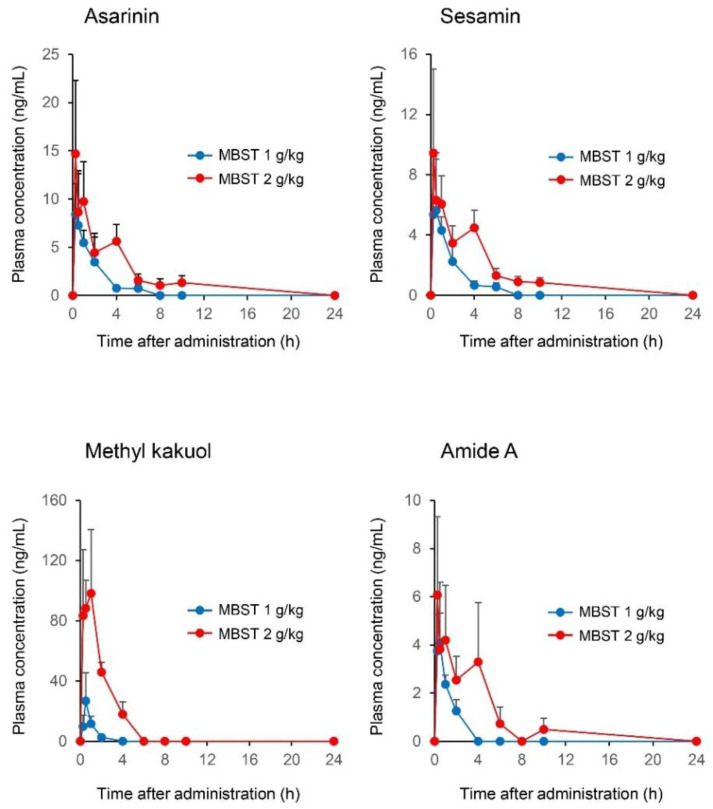
The plasma concentration-time profiles of asarinin, sesamin, methyl kakuol, and amide A ((2*E*,4*E*,8*Z*,10*E*)-*N*-isobutyl-2,4,8,10-dodecatetraenamide) derived from Asiasari Radix. Each plasma sample was obtained from whole rat blood after oral dosing of maobushisaishinto (MBST) at 1 or 2 g/kg dose and was analyzed via LC-MS/MS system using selected reaction monitoring mode. The MBST of 1 g/kg contains 578 µg/kg, 147 µg/kg, 226 µg/kg, 426 µg/kg asarinin, sesamin, methyl kakuol, and amide A, respectively. Individual points represent the mean + standard deviation for three independent animals. The pharmacokinetic analyses of the compounds were performed by a non-compartmental analysis (calculated pharmacokinetic parameters are shown in Table 2).

As shown in Figure 2 and Table 2, the maximum concentration (*C*_max_) of methyl kakuol at a dose of 2 g/kg (containing 1156 µg/kg, 294 µg/kg, 452 µg/kg, 852 µg/kg asarinin, sesamin, methyl kakuol, and amide A, respectively) was highest with 98.2 ng/mL, followed by asarinin. The epimeric furofuran lignan asarinin is a characteristic marker for AR quality. The asarinin content contained within MBST was predictably the highest among the AR ingredients, but the *C*_max_ value was lower than that of methyl kakuol. To the best of our knowledge, this is the first report to reveal a high plasma concentration of methyl kakuol following oral dosing of MBST. The plasma concentration-time profile of methyl kakuol indicated the characteristics of both rapid absorption and elimination. This study also revealed plasma concentrations of amide A using a standard substance chemically synthesized herein. Although the concentrations of amide A were relatively low, some alkylamides sharing a similar structural formula with amide A were detected in the non-targeted LC-HRMS analysis (Table 1). The additive or synergistic effect of the alkyl amides is speculated to be exerted in MBST treatment.

The *C*_max_ and area under the plasma concentration-time curve from zero to final observation time (AUC_0-last_) of all compounds were increased in a dose-dependent manner (Figure 2 and Table 2). Meanwhile, the plasma concentration-time curves of asarinin, sesamin, and amide A showed bimodality or trimodality at the 2 g/kg dose group but not at the 1 g/kg dose group (Figure 2). Unfortunately, it remains unclear why this phenomenon only occurred at a high dose of MBST. Some pharmacokinetic studies focusing on flavonoid aglycons and glycosides have reported bimodal or trimodal plasma concentrations of aglycon and refer to the involvement of enterohepatic circulation and the time required for metabolism from glycoside to aglycon by enterobacterium [43,44]. In the plasma concentrations of methyl kakuol, the degrees of increase in *C*_max_ and AUC_0-last_ were not proportional to the dosage increases in MBST (Figure 2 and Table 2). Because multi-ingredient drugs sometimes undergo self-pharmacokinetic interactions (e.g., modification of absorption or metabolism ratio) among their ingredients [45], it is speculated that other co-existing ingredients affected the pharmacokinetics of methyl kakuol at a high dose of MBST. To address the mechanisms of the non-linear pharmacokinetics, future studies, e.g., the verification of the pharmacokinetics following the sole administration of methyl kakuol, are needed.

### 2.3. Bioavailable Ingredients Derived from AR Did Not Affect Excessive NO Production That Is Involved in Inflammation

As described in the Introduction, MBST is widely used to care for the symptom caused by acute inflammatory diseases such as the common cold. In the disease, excessive inflammation is associated with the worsening of symptoms, and immune cells (e.g., macrophage) are deeply related to the response. The anti-inflammatory effects of MBST were observed in clinical situations. This effect can be explained by the actions of active ingredients in MBST especially those of Ephedrae Herba. For example, ephedrine and pseudoephedrine have been reported to have anti-inflammatory effects by suppressing the inflammatory cytokine tumor necrosis factor-α [46]. Several types of flavonoids, such as catechins, are well known reactive oxygen species scavengers [47]. In this study, we evaluated the effects of the bioavailable AR ingredients on the inflammatory response using a macrophage-like cell line, RAW264.7, and measured the NO production as an important factor for the modulation of the inflammatory response [48]. As the typical inducer of an inflammatory response by pathogen-associated molecular patterns, we used LPS, which is a ligand of toll-like 4 receptors. As mentioned below, AR ingredients have an activation effect against TRPA1. Although the activation of TRPA1 is known to possibly lead to inflammation, the previous article has suggested that TRPA1 agonist cannabichromene exerts anti-inflammatory actions via the inhibition of excessive nitric oxide [49]. Furthermore, sesamin and amide A were found to be TRPA1 agonists in this study and were reported to have an inhibitory effect on LPS-induced NO production in BV-2 microglial cells [29]. Therefore, we evaluated the inhibitory effect of AR ingredients against NO production. As for the results, we found that the test compounds did not affect LPS-induced NO production at concentrations of 1 or 10 µmol/L (see Supplementary Materials). This result suggests that AR ingredients may not participate in the anti-inflammatory effects of MBST, at least via inhibition of NO production.

### 2.4. Pharmacological Evaluation against TRP Channels of Bioavailable Ingredients Derived from MBST

In this pharmacological evaluation, TRP channels were selected based on reports that AR components have a pungent flavor upon human intake of AR [34], and that some herbal medicine-derived pungent components (e.g., 6-shogaol and cinnamaldehyde) have the potential to activate TRP channels [35]. TRP channels are important mediators of sensory signals that cause marked effects upon cellular functions and signaling pathways and represent promising druggable targets. Moreover, a recent study has shown that the activity of TRPA1 may contribute to T cell activation [50]. However, the effects of AR and MBST against TRP channels remain unclear. Therefore, we performed a Ca^2+^ influx assay to confirm the agonistic effect on several subfamilies of TRP channels—not only employing the bioavailable ingredients of AR, but also those of Ephedrae Herba and Aconiti Radix Processa—using stable cell lines expressing TRPA1, TRPV1, TRPV4, and TRPM8. As shown in Table 3, none of the tested compounds increased TRPV1-, TRPV4-, and TRPM8-mediated Ca^2+^ influx, but AR ingredients potently increased TRPA1-mediated Ca^2+^ influx at the same level of allyl isothiocyanate (AITC; used as positive control). As a result of the dose-response test, methyl kakuol showed the lowest half-maximal effective concentration (EC_50_) against TRPA1 at 0.27 µmol/L. Until now, the effect(s) of methyl kakuol, amide A, asarinin, and sesamin against TRP channels were unreported; thus, this is the first study to describe the agonistic effect of these compounds to TRPA1. TRPA1 is expressed in a set of nociceptive and thermoreceptive neurons and acts as a mediator of inflammatory pain generated by either noxious cold or chemical irritants [51,52]. TRPA1 activation on sensory neurons is known to exert vasodilation, leading to improved blood flow [53]. Additionally, the TRPA1 agonist AITC plays a role in enhancing thermogenesis and inhibiting heat diffusion [54]. Sahoo et al. (2019) revealed that TRPA1 is predominantly expressed at the surface of T cells and is involved in its activation [50]. Taken together, these results suggest that AR ingredients exert several actions via TRPA1 activation including vasodilation, adjuvanticity, and increases in body temperature, and these effects presumably contribute to the effects of MBST. Among AR ingredients, methyl kakuol most likely represents the active ingredient for TRPA1 activation because it has the lowest EC_50_ value and highest *C*_max_ following oral dosing of 2 g/kg MBST to rats. However, a clinical pharmacokinetic study is essential for understanding the accurate active ingredients in humans. Since we are planning a clinical pharmacokinetic study regarding MBST, we will report the results regarding human pharmacokinetics of AR ingredients in the future.

As described above, previous clinical studies report that MBST has adjuvanticity. Our current study has revealed that MBST ingredients, especially AR ingredients, show agonistic effects at TRPA1, which presumably leads to enhanced immune function via T cell activation. However, we did not directly evaluate T cell activation by MBST ingredients and did not investigate the effect on other immune functions such as macrophage function. Therefore, further pharmacological studies are required to reveal the detailed mechanisms of action of MBST.

## 3. Materials and Methods

### 3.1. Drugs and Reagents

Maobushisaishinto extract powder (lot no. 341100500), the base powder of MBST without excipients, was supplied from Tsumura & Co. (Tokyo, Japan), which manufactures it by spray-drying a hot water extract mixture of the three medical plants as follows (percent composition is shown in parentheses): the stem of *E. sinica* Stapf, *E. intermedia* Schrenk et C. A. Meyer, or *E. equisetina* Bunge (50%), tuberous roots of *A. carmichaeli* Debeaux or *A. japonicum* Thunberg (12.5%), and root and rhizome of *A. sieboldii* F. Maekawa or *A. heterotropoides* F. Maekawa var. *mandshuricum* F. Maekawa (37.5%). The quality was standardized and guaranteed by measuring several characteristic marker ingredients based on good manufacturing practice as defined by the Ministry of Health, Labor, and Welfare. AR extract powder (lot no. 2151026010) was also produced by spray-drying a hot water extract of the root and rhizome of *A. sieboldii* F. Maekawa or *A. heterotropoides* F. Maekawa var. *mandshuricum* F. Maekawa and supplied from Tsumura & Co.

(−)-Asarinin, neoline, ignavine, benzoylaconitine, methylephedrine N-oxide, catechin, epicatechin, and epigallocatechin were supplied by Tsumura & Co. (Tokyo, Japan). (1*R*,2*S*)-(−)-Ephedrine, (*S*,*S*)-(+)-pseudoephedrine, (1*R*,2*S*)-(−)-*N*-methylephedrine, niflumic acid, GSK1016790A, and icilin were obtained from Sigma-Aldrich (St. Louis, MO, USA). Sesamin, hippuric acid, AITC, and capsaicin were obtained from Fujifilm Wako Pure Chemical Industries (Osaka, Japan). Isovitexin and vicenin-2 were obtained from ChemFaces (Wuhan, China). Methyl kakuol (purity, 98.0% of HPLC total peak area) and amide A (purity, 82.9% of HPLC total peak area) were organically synthesized and supplied by Fuji Molecular Planning Co. (Yokohama, Japan) and Tsumura & Co. All other chemicals were purchased from commercial sources.

### 3.2. Animals

Seven-week-old male Sprague-Dawley rats were purchased from Charles River Laboratories (Yokohama, Japan) and Japan SLC (Hamamatsu, Japan). The animals were housed under a temperature of 23 °C ± 3 °C and relative humidity of 50% ± 20%, with 12-h/12-h light/dark cycle and allowed free access to water and standard laboratory food (MF, Oriental Yeast Co., Ltd., Tokyo, Japan). The animals were used in experiments following habituation for several days.

Ethics concerning the animal study were as follows: planning was made according to the guidelines for animal care and use of laboratory animals and was approved by the Institutional Animal Care and Use Committee (IACUC) of Tsumura & Co. (approval nos. and date, 16-024/July 14, 2016; 17-050/October 26, 2017).

### 3.3. Test Drug Doses in Rats

Extract powders of AR or MBST were suspended in distilled water and orally administered to rats having fasted for approximately 16 h. The dose of AR was set at 2 g/kg, and the MBST doses were set at 1 and 2 g/kg. 1 g/kg MBST contained 578 µg/kg, 147 µg/kg, 226 µg/kg, 426 µg/kg asarinin, sesamin, methyl kakuol, and amide A, respectively. The doses were chosen based on a previous pharmacological study of MBST using rats [60].

### 3.4. Plasma Sample Collection Following AR Administration

One animal (n = 1) at a time was sacrificed 1 h post-drug administration under anesthesia with isoflurane by collecting whole blood from the abdominal inferior vena cava using an EDTA-2K treated syringe. According to previous pharmacokinetic studies of herbal medicines [33,58], many of the components were quickly absorbed into the bloodstream, thereby providing the rationale for a sampling time at 1 h. Blood was centrifuged at 1200× *g* at 4 °C for 30 min to obtain plasma; then, the sample was stored at −80 °C until LC-HRMS analysis.

### 3.5. Plasma Sample Collection Following MBST Administration

The animals (n = 3/time point) were sacrificed at 0.25, 0.5, 1, 2, 4, 6, 8, 10, or 24 h under anesthesia with isoflurane after drug administration by collecting whole blood from the abdominal inferior vena cava using heparinized syringes. Blood samples were centrifuged at 1700× *g* at 4 °C for 15 min to obtain plasma, of which samples were then stored at ≤−75 °C until analysis.

Blank plasma samples were also obtained from normal rats without drug administration after fasting for approximately 16 h using the same procedure. These samples were used to generate the calibration curve for measuring plasma concentrations of AR ingredients in the analysis.

The plasma samples were collected at 1 h after MBST administration in case of both the qualitative (non-targeted) analysis and the quantitative (targeted) analysis.

### 3.6. Extract Sample Collection for the Quantification of the Contents of AR Ingredients in MBST

Four milliliters of a 75% methanol solution (*v*/*v*) was added to 100 mg of MBST extract powder and then vortexed and sonicated for 5 min each. The resultant solution was centrifuged at 1700× *g* at room temperature for 5 min, and the supernatant was collected. The residuum was treated repeatedly with 4 mL of 50% methanol solution (*v*/*v*), and approximately 8 mL of total mixed solution was obtained as extract sample for the quantification of the contents of AR ingredients in MBST. The extracted sample was diluted using a 5 mmol/L ammonium acetate solution, and was subsequently analyzed by the targeted analysis mentioned below.

### 3.7. Qualitative (Non-Targeted) Analysis of AR Components in Plasma after AR or MBST Administration

The plasma samples of AR- or MBST-treated rats collected at 1 h post-administration were pretreated by deproteinization using methanol before the LC-HRMS analysis as follows: 100 µL plasma sample was mixed with 500 µL methanol, and the resultant solution was vortexed for 2 min. The mixture was centrifuged at 7000× *g* at 4 °C for 5 min. The supernatant was dried using a centrifugal evaporator, and the dried residue was dissolved in 100 μL of the initial LC mobile phase which is composed of 0.1 vol% formic acid in water/acetonitrile (99.9/0.1, *v*/*v*) and recentrifuged at 7000× *g* at 4 °C for 5 min. Finally, a 5 μL aliquot of the supernatant was injected into the LC-HRMS system comprised of an Acquity Ultra-Performance Liquid Chromatography System (Waters, Milford, MA, USA) and a Xevo G2-XS quadrupole time-of-flight MS (Waters) and was chromatographically separated using an ACQUITY UPLC HSS T3 column (2.1 × 150 mm I.D., 1.8-μm particle size; Waters) with an ACQUITY UPLC HSS T3 VanGuard Pre-column (2.1 mm × 5 mm I.D., 1.8-μm particle size; Waters) at 40 °C. The mobile phase consisted of solution A (0.1% formic acid, *v*/*v*) and solution B (acetonitrile) with a gradient of solution B (0.1%, 0 min; 99%, 24 min; *v*/*v*) at a flow rate of 0.4 mL/min. The analysis of the mass spectrometer fit with an electrospray ionization probe with sensitivity mode was performed for both the positive and negative ion mode. The resolution of the mass spectrometer was set at >20,000. For MS^E^ experiments, two acquisition functions with different collision energies were created: a low-energy function with a collision energy of 6 eV and a high-energy function with collision energy ranging from 15 to 40 eV.

The matrix effects on the non-targeted analysis of methyl kakuol and amide A in the plasma were evaluated by the comparison of the peak areas between the sample of their standard substances dissolved in 0.1 vol% formic acid in water/acetonitrile (99.9/0.1, *v*/*v*) as well as a sample of blank plasma extract added to that (the final concentrations were same in both samples). The blank plasma extract was prepared by the same procedure with the pretreatment method mentioned above.

### 3.8. Quantitative (Targeted) Analysis of AR Components in Rat Plasma after MBST Administration

To quantify the plasma concentration of the AR components identified by the non-targeted analysis, the plasma samples at 0–24 h after MBST administration (1 or 2 g/kg) were supplied to targeted LC-MS/MS analysis in SRM mode with electrospray ionization. In the targeted analysis using triple quadruple MS (TripleQuad6500; SCIEX, Framingham, MA, USA) equipped with LC system (Agilent 1290 Infinity; Agilent Technologies, Santa Clara, CA, USA), we firstly preliminarily examined the optimal pretreatment and analytical LC-MS/MS methods (e.g., MS parameters and LC conditions) for the highly selective and sensitive detection of each compound.

For the quantification of methyl kakuol, amide A, asarinin, and sesamin in plasma, 100 µL of MBST-treated rat plasma was mixed with 20 µL of methanol (as the vehicle of the working solution described below) and 20 µL of internal standard niflumic acid (2 or 20 ng/mL). To prepare the calibration curve, the same volumes of blank plasma and various concentrations of working solution (final plasma concentration, 0.02–200 ng/mL) were used instead of the MBST-treated rat plasma and methanol. Finally, 500 µL of methanol was added for deproteinization. The mixture was centrifuged at 7000× *g* at 4 °C for 5 min. The supernatant was dried using a centrifugal evaporator. The dried residue was dissolved in 60 or 80 μL of the initial LC mobile phase which was composed of 5 mmol/L ammonium acetate in water/methanol (70/30, *v*/*v*), and then, a 5 or 10 μL aliquot was injected into the LC-MS/MS system. The injected sample was chromatographically separated using a YMC-Pack ODS-AQ column (2.0 ×150 mm I.D., 3-μm particle size; GL Sciences, Tokyo, Japan) at 30 °C. The mobile phase consisted of solution A (5 mmol/L ammonium acetate) as well as solution B (methanol), with a gradient of solution B (30%, 0 min; 70%, 25 min; 95%, 25.01–30 min; 30%, 30.01–35 min; *v*/*v*) at a flow rate of 0.3 mL/min. The MS/MS conditions are summarized in Table S2. In brief, the compounds were analyzed using optimal MS/MS parameters as follows: methyl kakuol (*m*/*z* 209.056/176.1), amide A (*m*/*z* 248.174/57.1), asarinin (*m*/*z* 372.123/173.1), and sesamin (*m*/*z* 372.118/233.1). The calibration ranges for methyl kakuol, amide A, asarinin, and sesamin were 0.5–100 ng/mL, 0.5–200 ng/mL, 0.1–200 ng/mL, 0.1–200 ng/mL, respectively.

The AR ingredient levels in the plasma are presented as the mean + standard deviation. Pharmacokinetic parameters of plasma AR ingredients, such as the *C*_max_, AUC_0-last_, were calculated by non-compartmental analysis using the Phoenix WinNonlin software (Certara L.P., St. Louis, MO, USA). The concentration data were pooled per each time point; then, the mean concentration–time data were subjected to the non-compartmental analysis.

### 3.9. Pharmacological Study

#### 3.9.1. Construction of TRP Channel-Expressing Cells

Each full-length human TRPA1, TRPV1, TRPV4, and TRPM8 coding regions were introduced into a pcDNA4/TO vector (Thermo Fisher Scientific, Waltham, MA, USA) and transfected into T-REx-293 cells (Thermo Fisher Scientific) to obtain stable cell lines expressing human TRPA1, TRPV1, TRPV4, and TRPM8. Cells were cultured in DMEM with 10% FBS, 4 mmol/L L-glutamine, and penicillin/streptomycin.

#### 3.9.2. Ca^2+^ Influx Assay for Evaluation of TRP Channel Activity

Cells were suspended in medium with 1 µg/mL tetracycline and seeded in poly-d-lysine coated 96-well plate at 2.5 × 10^4^ cells/well. After overnight culture, the cells were used for Ca^2+^ influx assay.

The Ca^2+^ influx assay was performed using a FLIPR Calcium 5 Assay Kit (Molecular Devices, Sunnyvale, CA, USA) according to previously reported methods [61] with minor modifications. In brief, cells were incubated with 80 µL Ca^2+^-chelating dye dissolved in assay buffer at pH 7.4 (Hanks’ Balanced Salt Solution containing 20 mmol/L of *N*-2-hydroxyethylpiperazine-*N*’-2-ethanesulfonic acid) at room temperature for 30 min. The plates were assayed using a FlexStation 3 Microplate Reader (Molecular Devices). The fluorescence intensity was traced entirely for 80 s. The baseline of fluorescence was measured for 20 s before adding 20 µL of test compound (final concentration, 10 µmol/L). The response was expressed as percentage activation using the relative fluorescence units (RFU) (% activation = 100 × ((RFU_max_ − RFU_baseline_)/RFU_baseline_) − 100). Furthermore, the activities of each test compound against several subfamilies of TRP channels were represented as the relative enhancement of Ca^2+^ influx of each positive control, indicated as follows: 20 µmol/L of allyl isothiocyanate (an agonist of TRPA1), 2 µmol/L of capsaicin (an agonist of TRPV1), 4 µmol/L of GSK1016790A (an agonist of TRPV4), and 20 µmol/L of icilin (an agonist of TRPM8). The dose–response curve was fitted to the sigmoidal dose-response equation (top, the max response of each compound), and EC_50_ values were calculated using GraphPad Prism 7 software (GraphPad, San Diego, CA, USA).

## 4. Conclusions

This study employed a strategic approach to identify the bioavailable active ingredients and pharmacological roles of AR within MBST. The results suggest that methyl kakuol, amide A, asarinin, and sesamin are potential active ingredients within AR and MBST, as these were absorbed following the oral administration of MBST, and exhibited an agonistic effect against TRPA1. In particular, methyl kakuol showed the highest plasma concentration and a lowest EC_50_ value of TRPA1 activation, and was thought to be the most influential ingredient within AR, although further pharmacokinetic and pharmacological studies are needed.

## Figures and Tables

**Table 1:** Ingredients derived from Asiasari Radix were detected and identified in non-targeted analysis utilizing LC-HRMS.

*t*_R_ (min)	Adduct	Observed Intact Mass (Da)	Error (ppm)	Molecular Formula	Assigned and Estimated Compound from Our Chemical Database	AR-Treated Rat Plasma ^†^	MBST-Treated Rat Plasma ^†^	Characteristic Fragment Ions	Identification Results
6.87	+H	148.0884	−2.7	C_10_H_12_O	Estragole	+	ND	ND	NI
9.36	+H	281.2001	3.5	C_16_H_27_NO_3_	(2*E*,4*E*,9*E*)-8,11-Dihydroxy-*N*-isobutyl-2,4,9-dodecatrienamide, (2*E*,4*E*,8*Z*)-10,11-Dihydroxy-*N*-isobutyl-2,4,8-dodecatrienamide	+	+ + +	ND	NI
9.64	+H	281.2000	3.2	C_16_H_27_NO_3_	(2*E*,4*E*,9*E*)-8,11-Dihydroxy-*N*-isobutyl-2,4,9-dodecatrienamide, (2*E*,4*E*,8*Z*)-10,11-Dihydroxy-*N*-isobutyl-2,4,8-dodecatrienamide	+	+ + +	264.1963	NI
9.82	+H	281.1997	2.0	C_16_H_27_NO_3_	(2*E*,4*E*,9*E*)-8,11-Dihydroxy-*N*-isobutyl-2,4,9-dodecatrienamide, (2*E*,4*E*,8*Z*)-10,11-Dihydroxy-*N*-isobutyl-2,4,8-dodecatrienamide	+	+ + +	ND	NI
11.32	−H	357.0468	−4.7	C_17_H_11_NO_8_	Aristolochic acid Iva	+	+ + +	310.0463, 307.0452, 281.0461, 266.0571, 265.0475, 251.0328	NI
11.49	−H	342.1094	−2.8	C_19_H_18_O_6_	(7α,7′β,8α,8′α)-3,4-Methylenedioxy-3′,4′-dihydroxy-7,9′:7′,9-diepoxylignane	+	ND	311.0910, 176.0458, 175.0396	NI
12.43	+H	208.0734	−0.6	C_11_H_12_O_4_	Methyl kakuol	+	+ + +	191.0701, 179.0333, 176.0460, 165.0173	Methyl kakuol
15.05	+H	221.1780	0.3	C_14_H_23_NO	Spilanthol	+	+ + +	ND	NI
16.30	+H, +Na	247.1937	0.4	C_16_H_25_NO	Amide A	+	+ + +	167.1289, 166.1222	Amide B ^#^
16.39	+H, +Na	247.1937	0.2	C_16_H_25_NO	Amide A	+	+ + +	167.1298, 166.1221, 152.1040	Amide A
16.59	+H	247.1935	−0.5	C_16_H_25_NO	Amide A	+	+ + +	ND	*N*-Isobutyl-2,4,8,10-dodecatetraenamide isomer ^#^

*t*_R_, retention time; amide A, (2*E*,4*E*,8*Z*,10*E*)-*N*-isobutyl-2,4,8,10-dodecatetraenamide; amide B, (2*E*,4*E*,8*Z*,10*Z*)-*N*-isobutyl-2,4,8,10-dodecatetraenamide; ND, not detected; NI, not identified. From the assigned name obtained with our chemical database, the available standard substances were prepared and used for identifying the compound. ^#^: These compounds were not confirmed using an authentic standard substance but were tentatively identified based on the analytical information of a previous report [39]. ^†^: The number of plus signs represents the sample number in which the compound was detected among AR-treated rat plasma (n = 1) and MBST-treated rat plasma (n = 3). Plasma samples were obtained from whole rat blood 1 h following oral administration of 2 g/kg AR or MBST.

**Table 2:** Pharmacokinetic parameters of Asiasari Radix ingredients measured in maobushisaishinto (MBST)-treated rat plasma.

Compound	Dose of MBST (g/kg Body Weight)	Dose of Ingredient within MBST (µg/kg)	*C*_max_ (ng/mL)	*t*_max_ (h)	AUC*_0-last_* (ng·h/mL)	*t*_1/2_ (h)	*k*_el_ (h^−1^)
Asarinin	1	578	8.42	0.25	16.3	1.52	0.456
	2	1156	14.7	0.25	38.6	2.93	0.237
Sesamin	1	147	5.63	0.5	11.9	1.63	0.425
	2	294	9.44	0.25	28.7	2.99	0.232
Methyl kakuol	1	226	26.7	0.5	22.5	0.452	1.53
	2	452	98.2	1.0	214	1.26	0.552
Amide A	1	426	4.08	0.5	4.87	0.912	0.760
	2	852	6.07	0.25	18.4	2.92	0.237

*k*_el_: terminal elimination rate constant. Amide A, (2*E*,4*E*,8*Z*,10*E*)-*N*-isobutyl-2,4,8,10-dodecatetraenamide; *C*_max_, maximum concentration; *t*_max_, time to maximum concentration; AUC_0–last_, area under the plasma concentration-time curve from zero to final observation time; *t*_1/2_, apparent elimination half-life. All values were calculated by the mean concentration (n = 3) per time point with a non-compartmental analysis using the Phoenix WinNonlin software (Certara L.P., St. Louis, MO, USA).

**Table 3:** Agonistic effect of maobushisaishinto ingredients against TRP channels.

Crude Drug	Test Compound	% of Activation Compared to Positive Control against Each Subfamily of TRP Channel				EC_50_ Value against TRPA1 (µmol/L)	Pharmacokinetics Study Reference
		TRPA1	TRPV1	TRPV4	TRPM8		
Asiasari Radix	Methyl kakuol	49	0.1	0.9	2.8	0.27	Herein
	Amide A	96	0.7	2.7	6.1	0.47	Herein
	Asarinin	106	−0.4	−0.1	1.4	3.1	Herein
	Sesamin	106	−0.1	−0.1	−0.8	2.3	Herein
Aconiti Radix Processa	Neoline	5	−0.4	0.5	5.0	—	[27]
	Ignavine	2	−0.2	−0.1	1.8	—	[28]
	Benzoylaconitine	3	−0.2	0.0	3.6	—	[55]
Ephedrae Herba	Isovitexin	18	0.2	2.3	4.3	—	[56]
	Vicenin-2	25	1.8	5.0	6.5	—	[57]
	Ephedrine	3	−0.5	−0.4	3.2	—	[33]
	Pseudoephedrine	4	−0.2	−0.3	2.8	—	[33]
	Methylephedrine	3	−0.4	−0.1	3.6	—	[33]
	Methylephedrine N-oxide	21	0.3	1.8	4.5	—	[33]
	Catechin	6	−0.3	0.3	4.0	—	[58]
	Epicatechin	6	0.0	0.6	4.2	—	[59]
	Epigallocatechin	2	−0.2	0.1	4.8	—	[59]
	Hippuric acid	13	1.0	2.8	7.5	—	[33]

Amide A, (2*E*,4*E*,8*Z*,10*E*)-*N*-isobutyl-2,4,8,10-dodecatetraenamide; —, not determined. Calcium ion influx into TRPA1-, TRPV1-, TRPV4-, or TRPM8-expressing T-REx-293 cells was measured to evaluate the agonistic effect on TRP channels of MBST ingredients. The action of 10 µmol/L of each test compound was represented as the relative enhancement of Ca^2+^ influx of each positive control, indicated as follows: 20 µmol/L allyl isothiocyanate (an agonist of TRPA1), 2 µmol/L capsaicin (an agonist of TRPV1), 4 µmol/L GSK1016790A (an agonist of TRPV4), and 20 µmol/L icilin (an agonist of TRPM8). Dose-response tests of MBST ingredients against TRPA1 were performed to calculate the EC_50_ value. Each test compound was selected based on reported pharmacokinetic information.

## Supplementary Materials

The following are available online, Figure S1: Structural formulas of eight compounds estimated to be AR-derived ingredients within the non-targeted analysis of plasma harvested from AR- or MBST-treated rats, Figure S2: MS and MS/MS information used in the identification of methyl kakuol and (2*E*,4*E*,8*Z*,10*E*)-*N*-isobutyl-2,4,8,10-dodecatetraenamide via LC-HRMS analysis, Figure S3: Representative SRM chromatograms of methyl kakuol, amide A, asarinin, and sesamin in the blank plasma with a mixture of their standard substances, as well as blank plasma, and plasma collected at 1 h after the administration of 1 g/kg of MBST, Table S1: List of AR ingredients incorporated into UNIFI software, Table S2: Targeted LC-MS/MS methods: ion parameters of test compounds.
