# Supplementary material for: In Vivo Pharmacokinetic Analysis Utilizing Non-Targeted and Targeted Mass Spectrometry and In Vitro Assay against Transient Receptor Potential Channels of Maobushisaishinto and Its Constituent Asiasari Radix
Source: Molecules. 2020 Sep 18;25(18):4283. doi: 10.3390/molecules25184283 (PMC7570662; doi:10.3390/molecules25184283)
Supplement: Supplementary file 1 [file molecules-25-04283-s001.pdf]

Table S1. List of AR ingredients incorporated into UNIFI software.

(-)-Pipelitol	Hydroferulic acid
(+)-7'-Methoxylariciresinol	Isovanillic acid
(2*E*,4*E*,8*Z*)-10,11-Dihydroxy-N-isobutyl-2,4,8-dodecatrienamide	Kakuol
(2*E*,4*E*,8*Z*,10*E*)-N-Isobutyl-2,4,8,10-dodecatetraenamide	Methyl kakuol
(2*E*,4*E*,9*E*)-8,11-Dihydroxy-N-isobutyl-2,4,9-dodecatrienamide	Limonene
(7S,8R,7'S,8'S)-3-Methoxy-3',4'-methylenedioxy-7,9'-epoxylignane-4,7',9-triol	Linalool
(7α,7'β,8α,8'α)-3,4-Methylenedioxy-3',4'-dihydroxy-7,9':7',9-diepoxylignane	Methyleugenol
(R)-5-(2,3-Dihydroxypropyl)-1,3-benzodioxole	Methylpluviatilol
3,4,5-Trimethoxytoluene	Myrcene
3,4-Dimethoxybenzenepropionic acid	Myristicin
3,4-Methylenedioxy-propiophenone	Naringenin
3,5-Dimethoxytoluene	Naringenin-7-glucoside
3-Carene	N-Isobutyl-2,4,8,10,12-tetradecapentaenamide
4-Hydroxybenzoic acid	N-Isobutyl-2,4,8,10-tetradecatetraenamide
7-Methoxyaristololactam IV	N-Isobutyl-2,4,8-dodecatrienamide
Aristolactam I	N-Isobutyl-2,4-dodecadienamide
Aristolochic acid Iva	Pellitorine
Asaricin	Pentadecane
Asarinin	Phellandrene
Asarinol A	Pinene
Asarinol D	Sabinene
Asarone	Safrole
Borneol	Sesamin
Camphor	Spilanthol
Croweacin	Terpinen-4-ol
Cymen-8-ol	Terpineol
Elemene	Terpinolene
Elemicin	Tetradecane
Epipinoresinol	*trans*-*p*-Feruloyl-*β*-D-glucopyranoside
Estragole	Vanillic acid
Eucalyptol	Xanthoxylol
Eucarvone	Zingiberene

Table S2. Targeted LC-MS/MS methods: ion parameters of test compounds.

Compound name	Q1 mass	Q3 mass	DP	CE	CXP
	m/z	*m/z*	volts	volts	volts
Asarinin	372.123	173.1	6	25	10
Sesamin	372.118	233.1	11	11	14
Methyl kakuol	209.056	176.1	66	27	14
Amide A	248.174	57.1	1	29	10
Niflumic acid (internal standard)	283.100	265.1	101	31	20

Amide A, (2*E*,4*E*,8*Z*,10*E*)-N-isobutyl-2,4,8,10-dodecatetraenamide; DP, declustering potential; CE, collision energy; CXP, collision cell exit potential.

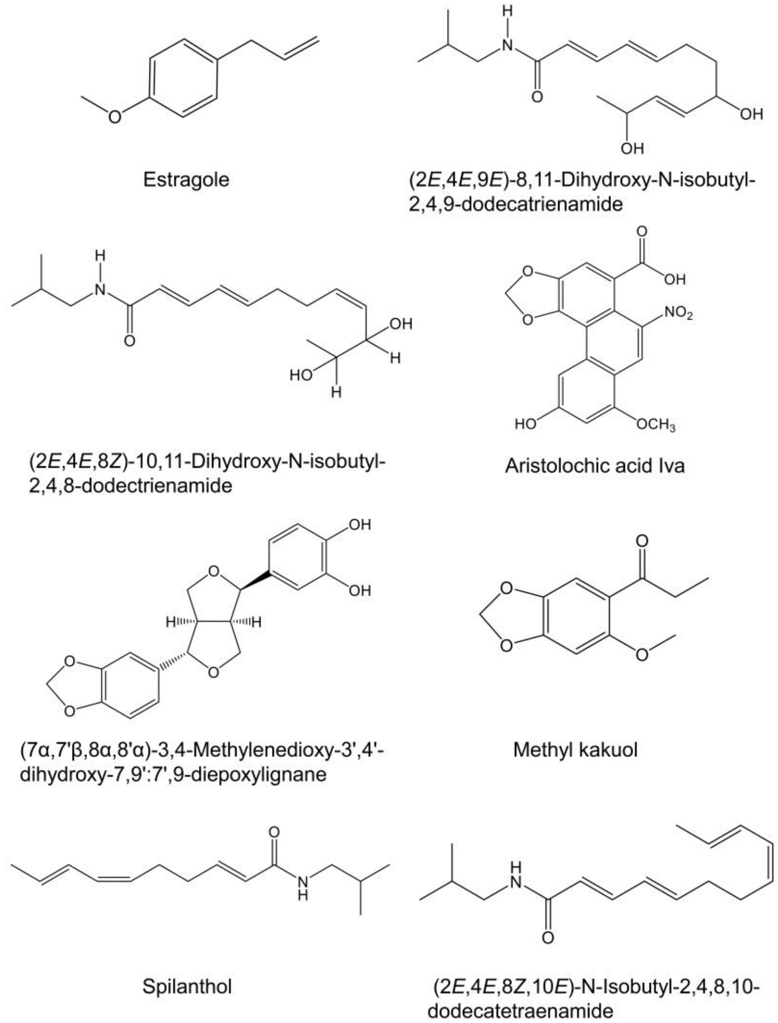

Figure S1: Structural formulas of eight compounds estimated to be AR-derived ingredients within the non-targeted analysis of plasma harvested from AR- or MBST-treated rats

Target compound: methyl kakuol

Analytical sample: standard substance of methyl kakuol

(Extract ion chromatogram of 209.07 Da)

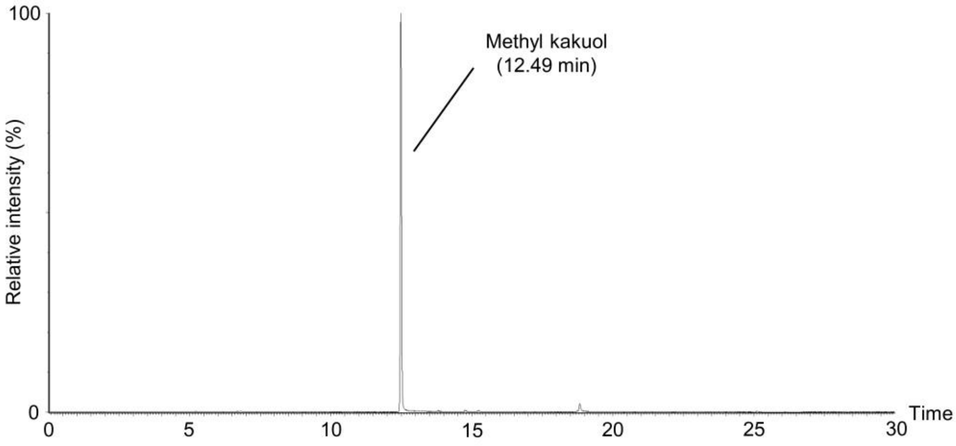

(MS^2 spectrum of peak detected at 12.49 min)

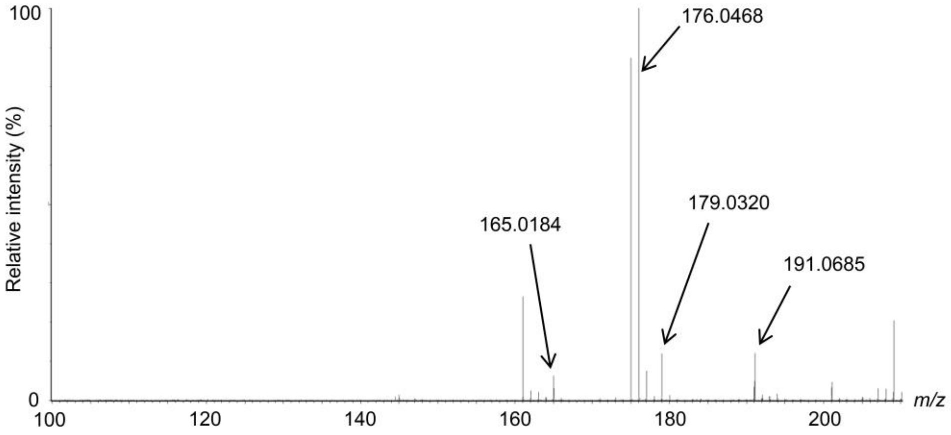

Target compound: methyl kakuol

Analytical sample: extract of maobushisaishinto

(Extract ion chromatogram of 209.07 Da)

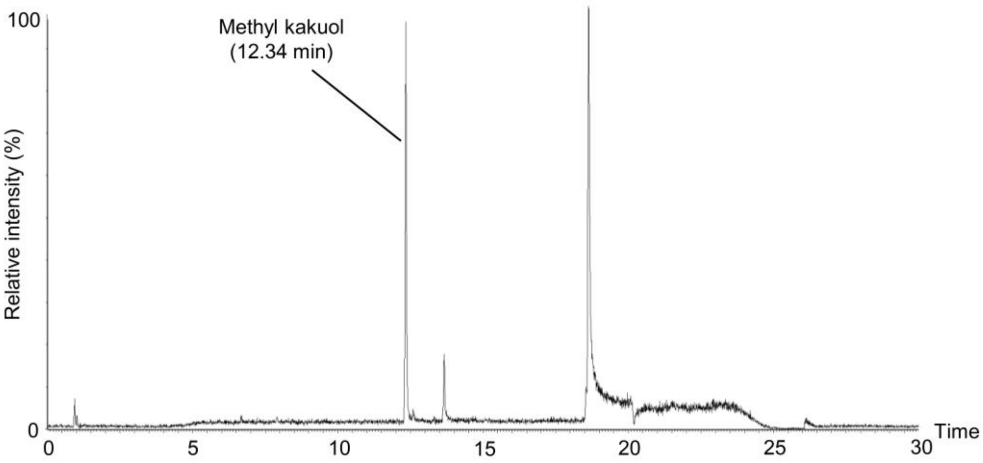

(MS^2 spectrum of peak detected at 12.34 min)

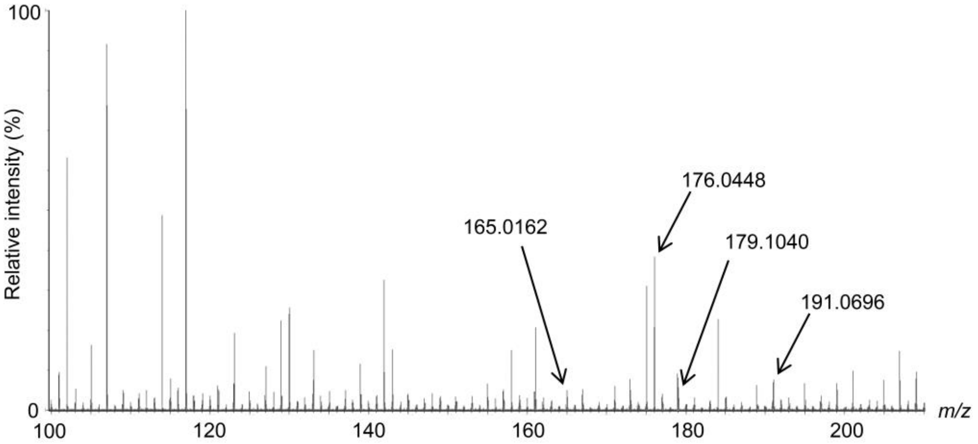

Target compound: methyl kakuol

Analytical sample: Asiasari Radix-treated rat plasma

(Extract ion chromatogram of 209.07 Da)

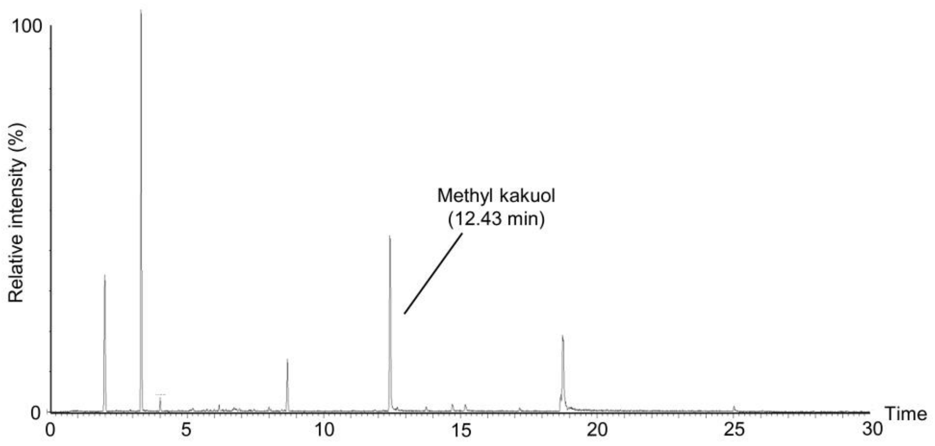

(MS^2 spectrum of peak detected at 12.43 min)

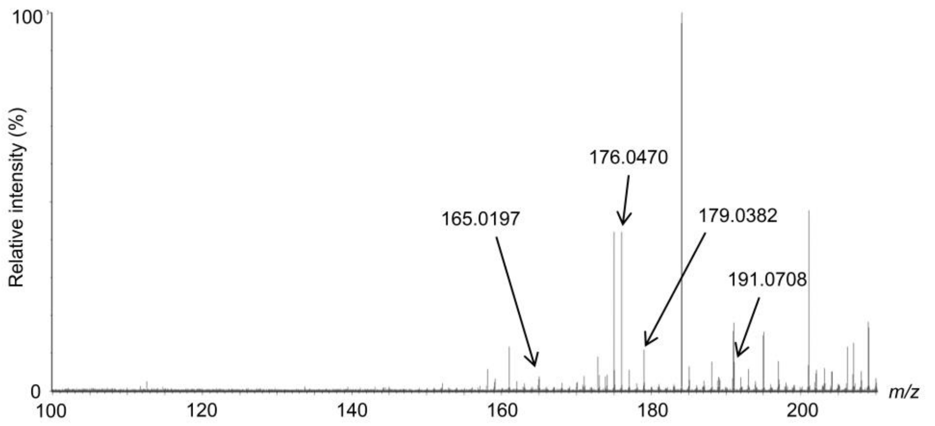

Target compound: (2*E*,4*E*,8*Z*,10*E*)-N-Isobutyl-2,4,8,10-dodecatetraenamide

Analytical sample: standard substance of

(2*E*,4*E*,8*Z*,10*E*)-N-Isobutyl-2,4,8,10-dodecatetraenamide

(Extract ion chromatogram of 248.20 Da)

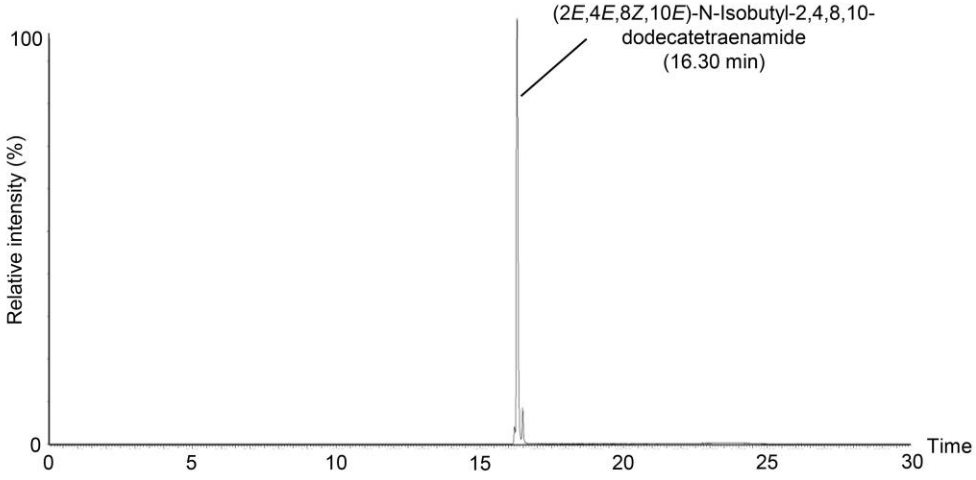

(MS^2 spectrum of peak detected at 16.30 min)

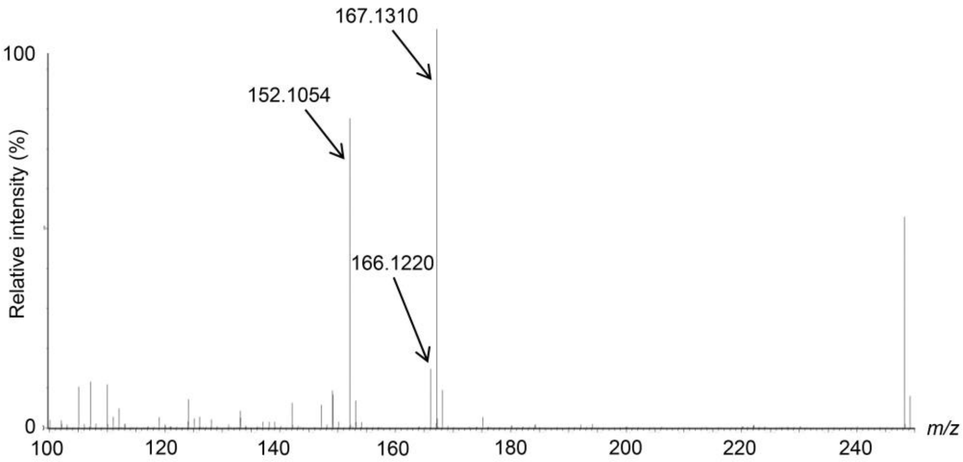

Target compound: (2*E*,4*E*,8*Z*,10*E*)-N-Isobutyl-2,4,8,10-dodecatetraenamide

Analytical sample: extract of maobushisaishinto

(Extract ion chromatogram of 248.20 Da)

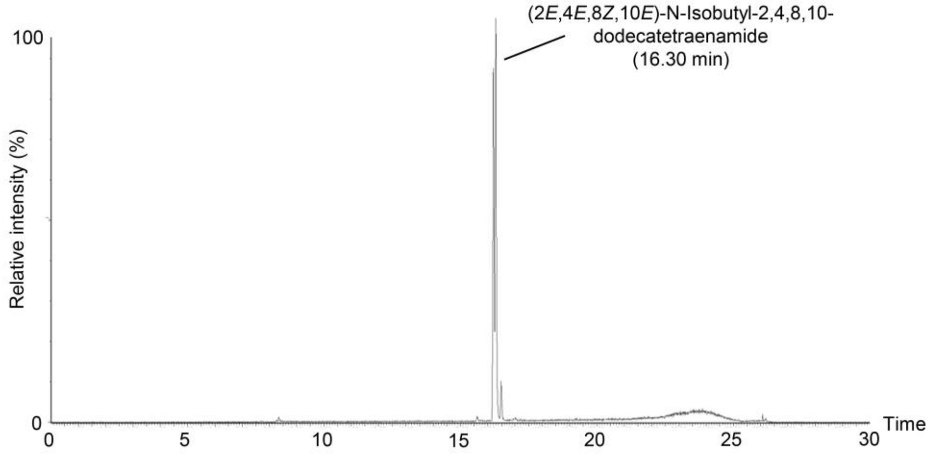

(MS^2 spectrum of peak detected at 16.30 min)

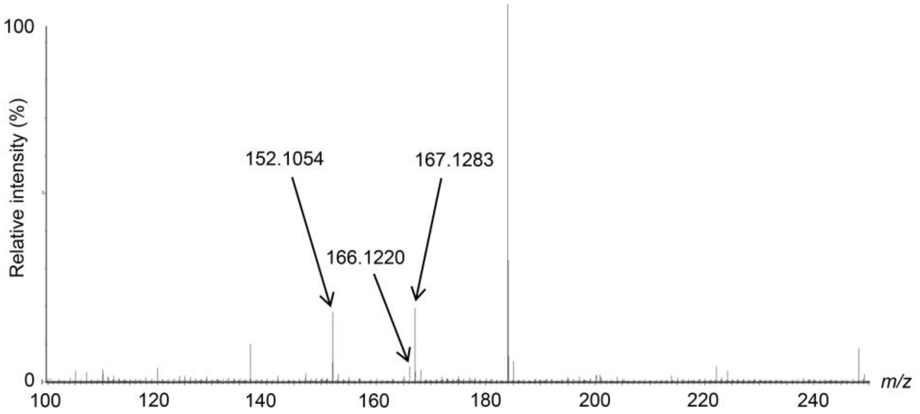

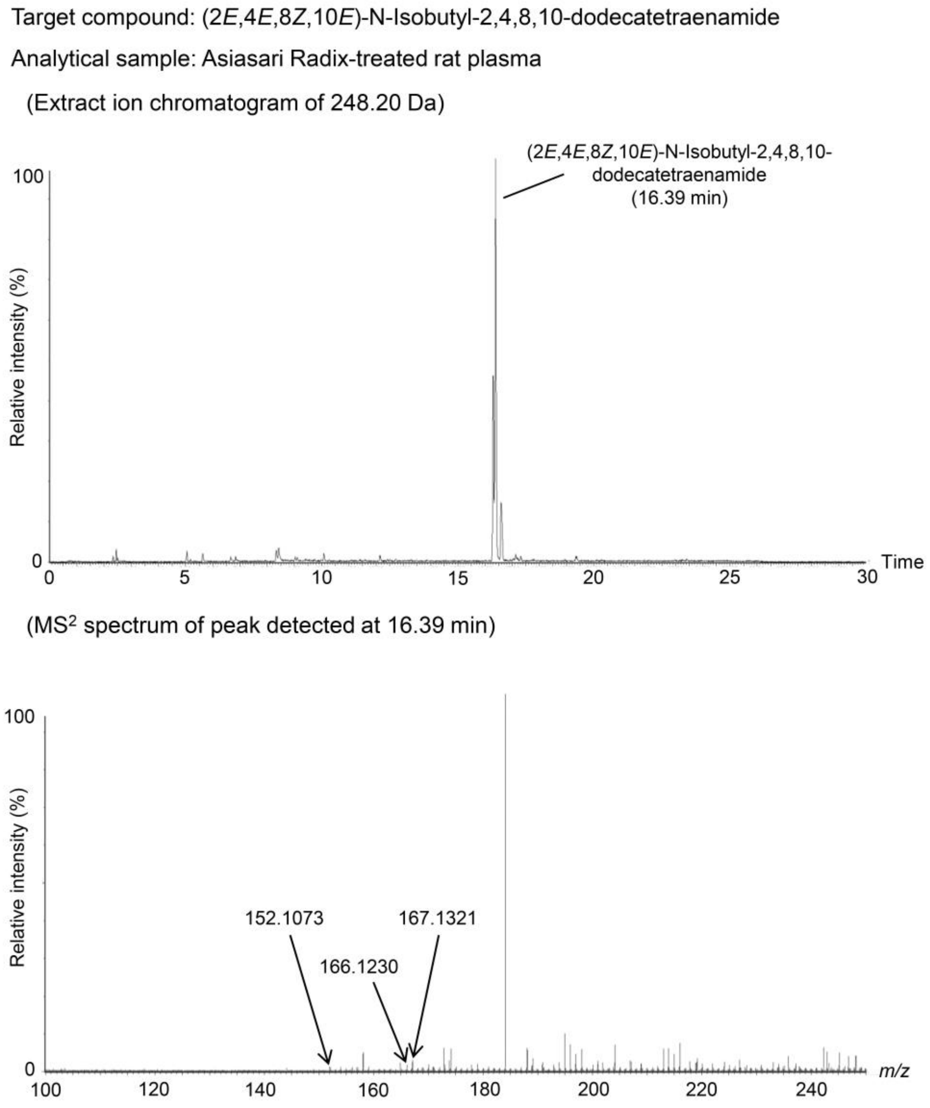

Figure S2: MS and MS/MS information used in the identification of methyl kakuol and (2*E*,4*E*,8*Z*,10*E*)-N-isobutyl-2,4,8,10-dodecatetraenamide via LC-HRMS analysis.

Target compound: methyl kakuol

(SRM chromatogram of *m/z* 209.056/176.1)

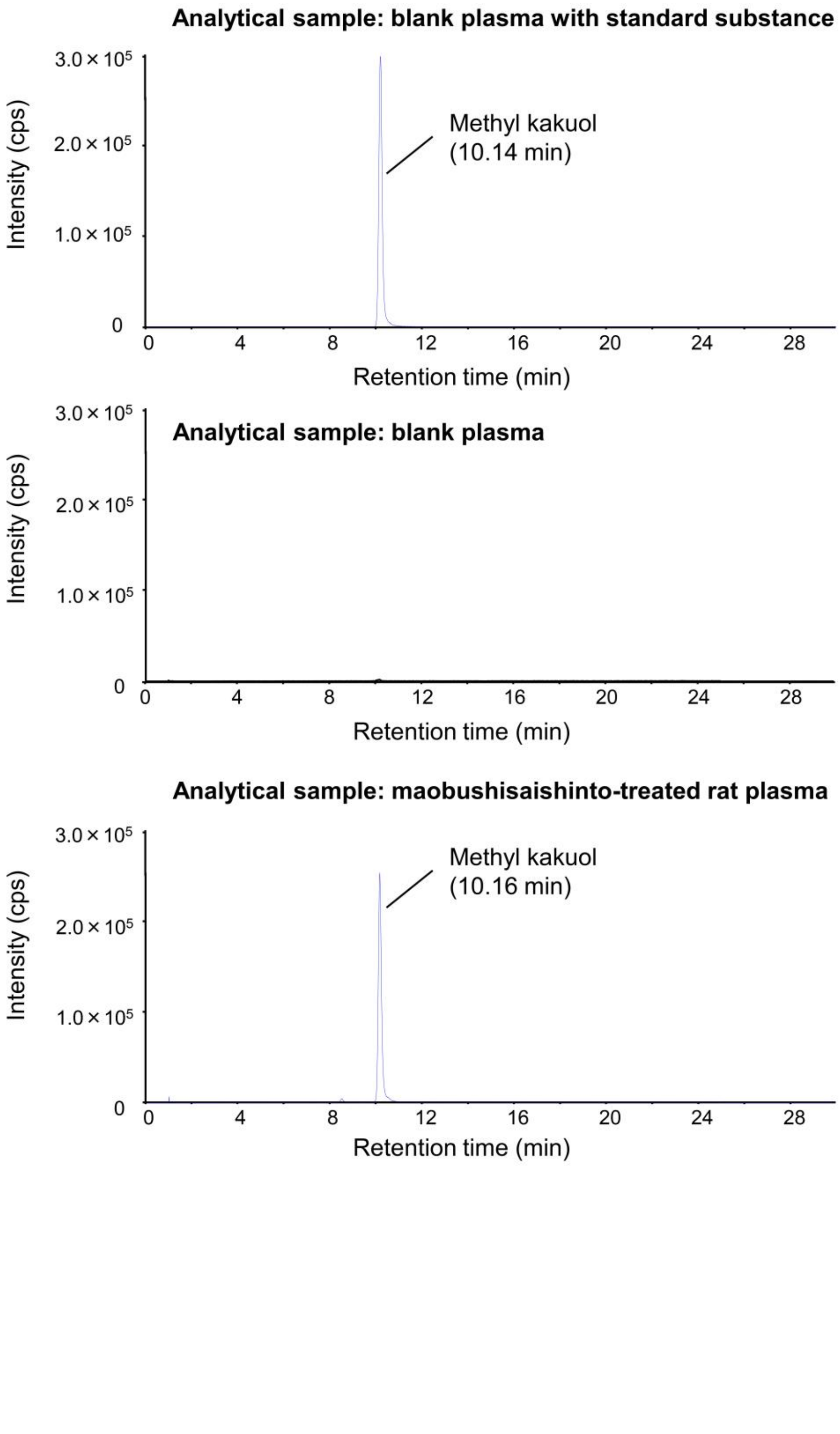

Target compound: (2*E*,4*E*,8*Z*,10*E*)-N-isobutyl-2,4,8,10-dodecatetraenamide (SRM chromatogram of *m/z* 248.174/57.1)

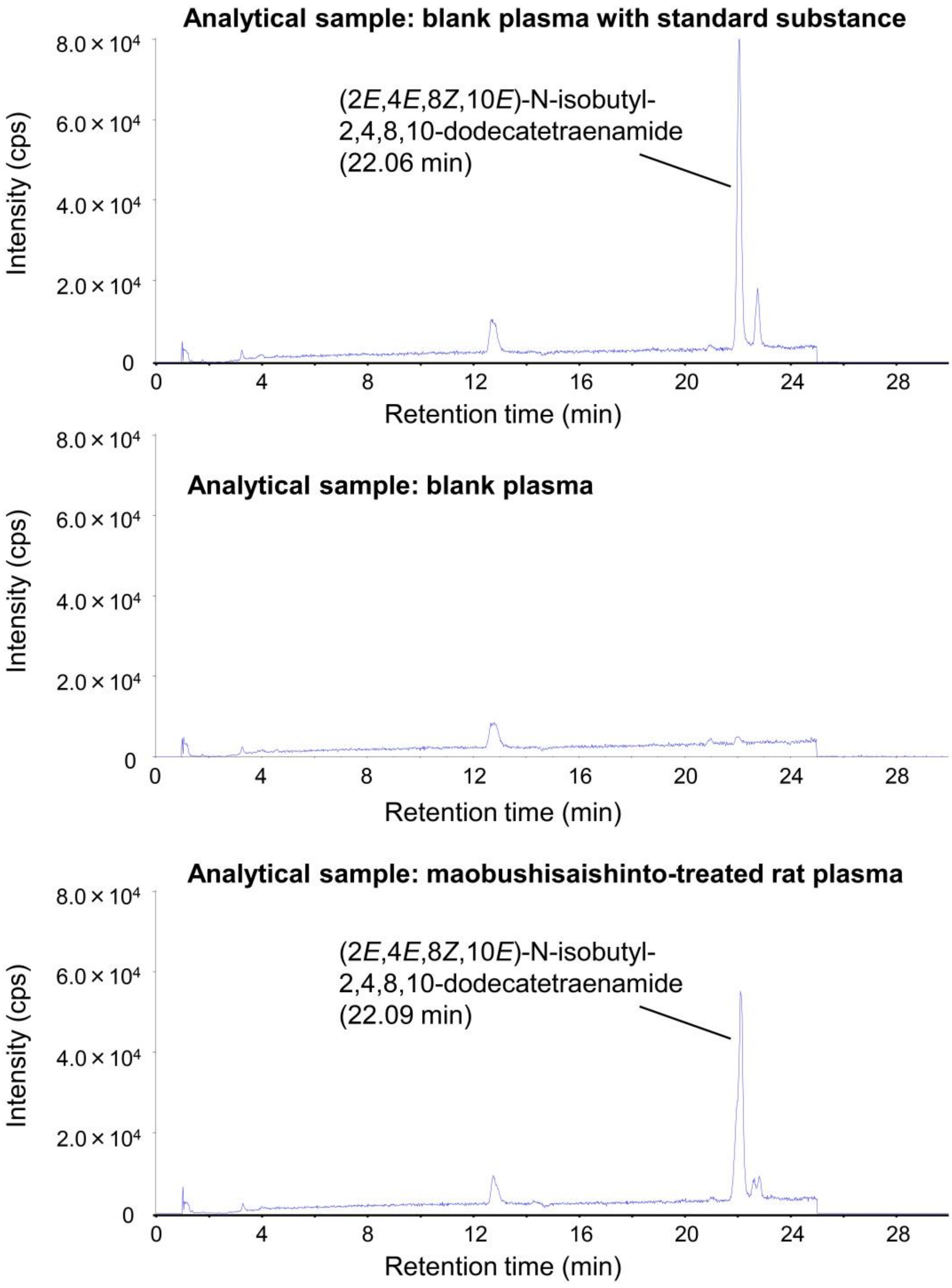

Target compound: asarinin

(SRM chromatogram of *m/z* 372.123/173.1)

Analytical sample: blank plasma with standard substance

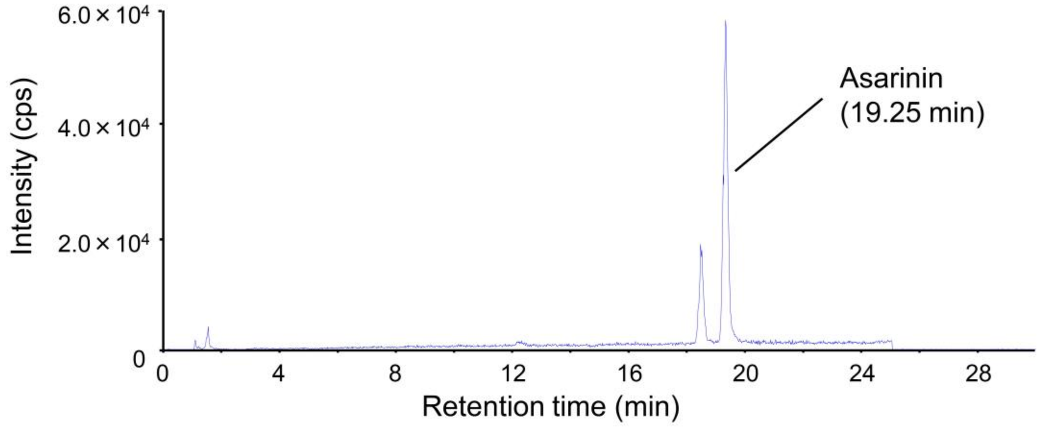

Analytical sample: blank plasma

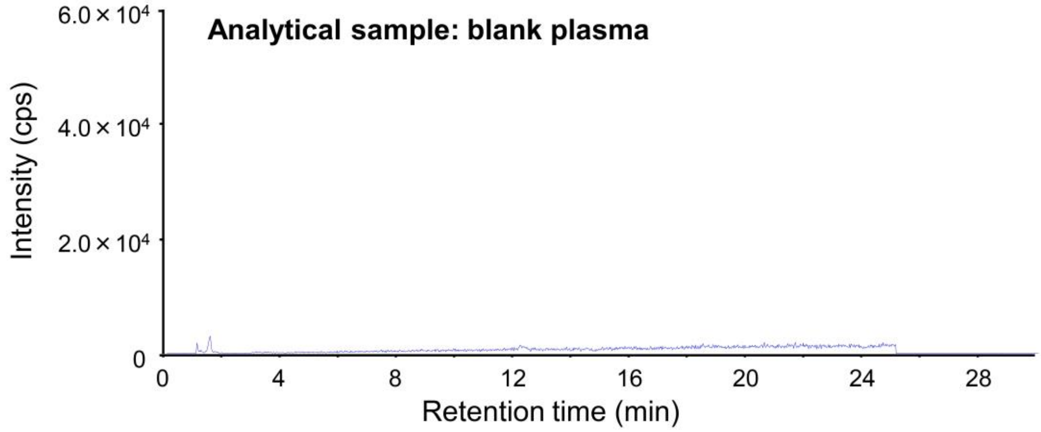

Analytical sample: maobushisaishinto-treated rat plasma

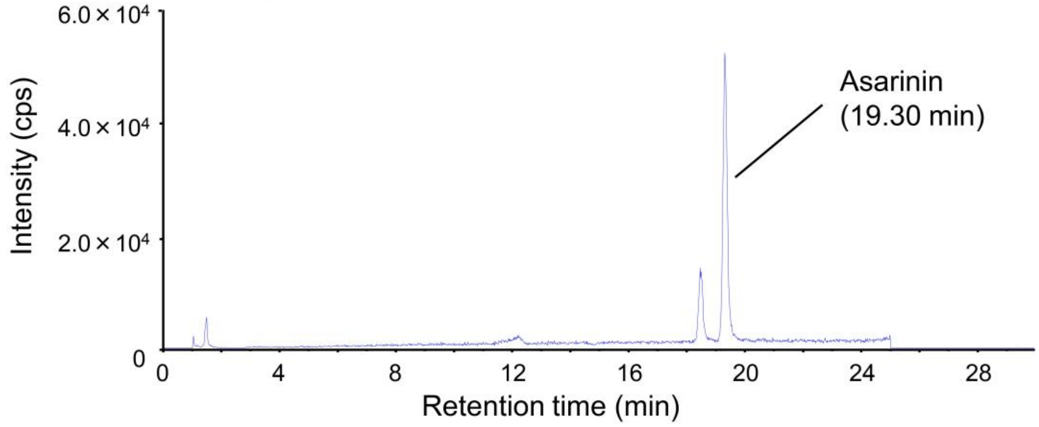

Target compound: sesamin

(SRM chromatogram of *m/z* 372.118/233.1)

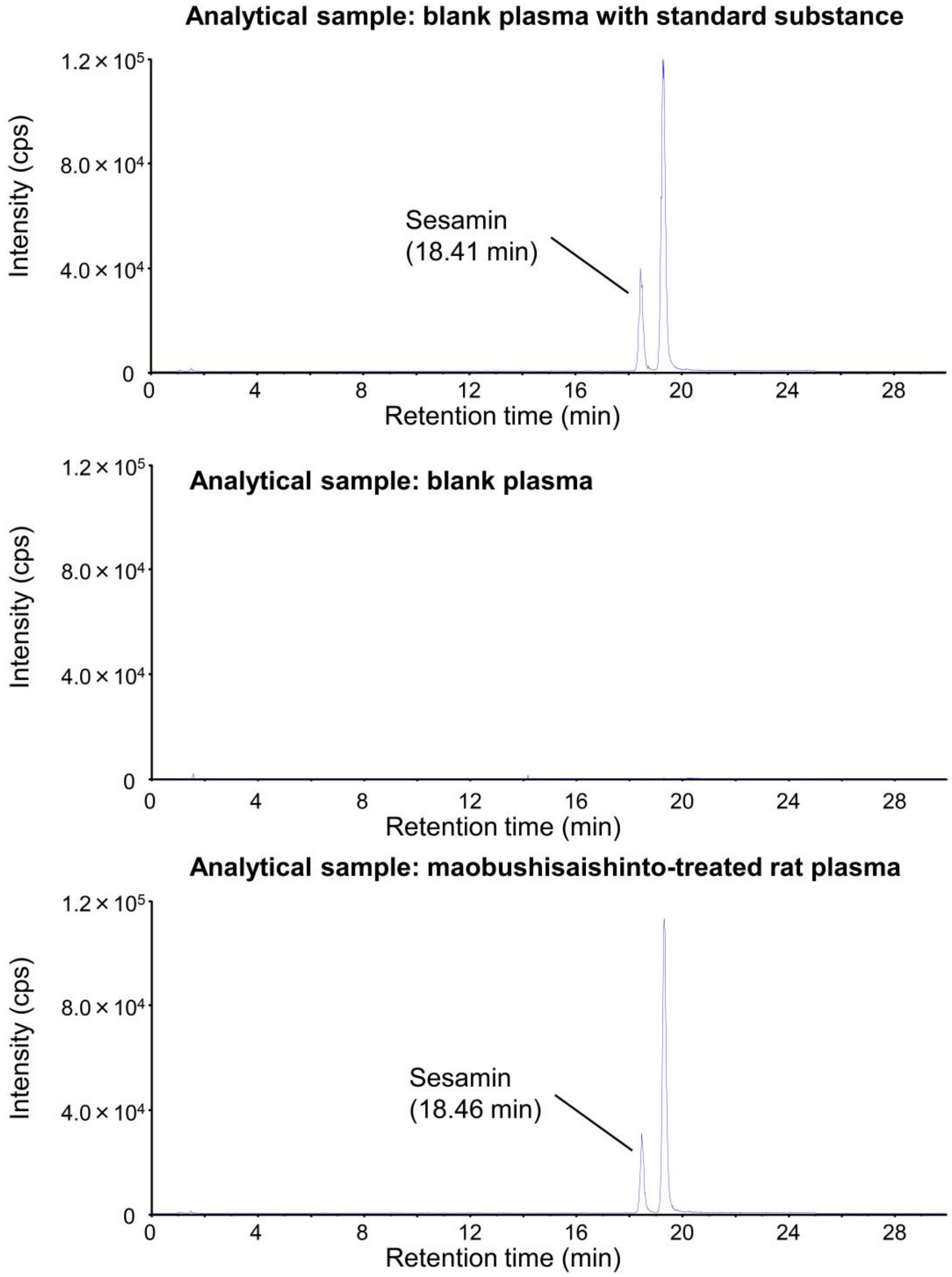

Figure S3: Representative SRM chromatograms of methyl kakuol, amide A, asarinin, and sesamin in the blank plasma with a mixture of their standard substances, as well as blank plasma, and plasma collected at 1 hour after the administration of 1 g/kg of MBST

The experimental methods and results about the measurement of extracellular NO production are described as following.

Methods

Mouse macrophage-like RAW264. 7 cells were obtained from the European Collection of Authenticated Cell Cultures and cultured in Dulbecco's Modified Eagle Medium (DMEM) containing 10% fetal bovine serum (FBS). The cells were maintained at 37 °C in a humidified incubator with an atmosphere of 5% CO_2.

RAW264. 7 cells were seeded in 48-well plates (6.0×10^4 cells per well) and incubated overnight, and then, 1 or 10 μmol/L of test compounds and 3 μg/mL of lipopolysaccharide (LPS; from *Escherichia coli* 0111: B4, L3024; Sigma-Aldrich) were added into the medium. Following 24 h after addition, 100 μL of conditioned media were collected and mixed with equal volume of Griess reagent (G4410; Sigma-Aldrich), and the mixture was incubated at room temperature for 15 min. Finally, absorbance was determined at 540 nm wave. Nitrite concentrations were quantified using a calibration curve obtained from a dilute solution of $NaNO_2$.

Results

As shown in Table S3, almost all of the test compounds did not affect LPS-induced NO production at concentrations of 1 or 10 μmol/L.

Table S3. Effect of Asiasari Radix ingredients on LPS-induced NO production.

**Test Compound**	**NO Production (% of LPS Control)**	
	**1 μmol/L**	**10 μmol/L**
Methyl kakuol	102	93
Amide A	92	82
Asarinin	99	97
Sesamin	96	78

Amide A: (2*E*,4*E*,8*Z*,10*E*)-*N*-isobutyl-2,4,8,10-dodecatetraenamide. Each test compound (1 or 10 μmol/L) and lipopolysaccharide (LPS, 3 μg/mL) were added into a culture of RAW264. 7 cells. Following 24 h of substance addition, nitric oxide (NO) released in the cell medium was measured by Griess method. Percentage of NO production was calculated using the following equation: (nitrite concentration after treatment with test compound and LPS/nitrite concentration after treatment with LPS only) × 100. Each value represents the mean of two independent experiments
